# Unconditional cash transfers for breastfeeding women in the Mexican informal workforce: Time for social justice

**DOI:** 10.3389/fpubh.2023.1101466

**Published:** 2023-03-01

**Authors:** Sofía Barragán-Vázquez, Jocelyn Jaen, Sonia Collado-López

**Affiliations:** ^1^Center for Nutrition and Health Research, National Institute of Public Health, Cuernavaca, Morelos, Mexico; ^2^School of Public Health of Mexico, National Institute of Public Health, Cuernavaca, Morelos, Mexico

**Keywords:** breastfeeding, health economics, social justice, informal economy, low-and middle-income countries, unconditional cash transfers

## 1. Introduction

Proper nutrition during early life is essential to ensure a child's adequate growth, health, and development, preventing morbidity and mortality in the short and long term (1–4). Human milk is the optimal food source during the first 6 months of life, it naturally changes its composition in function with the child's growth to fit their needs. However, the prevalence of exclusive breastfeeding (EBF) during the first 6 months of life has been considerably low in Mexico and many low-and middle-income countries (LMICs) (5).

Women's employment has been reported as a key determinant for not breastfeeding in different countries, and working women have lower probabilities of ever breastfeeding (6–8). This is partly due to inadequate maternity leave legislations, which only covers those in formal employment, as well as inadequate support from family and health services. Current Mexican breastfeeding legislation provides support only to formally working mothers (9). Women in the informal economy (which includes diverse economic activities, jobs, and workers that are not regulated or protected by the Government), despite representing more than half of the female working force in Mexico (10) and in other LMICs (11), have long been overlooked by maternity legislation and policies.

### 1.1. Exclusive breastfeeding in Mexico

Mexico is still far from the global nutrition target of Exclusive Breastfeeding (EBF) by 2025, and health inequities are still present. From 2006 to 2012, the National Health and Nutrition Survey (ENSANUT) in Mexico showed that the prevalence of EBF decreased from 22.3 to 14.4%, particularly in rural areas, causing a health and economic burden of ~3,000 million dollars per year (7, 12). Calculations included direct health care costs of the diseases associated with inadequate breastfeeding practices, lost future earnings due to premature death, and infant formula expenses.

Actions were taken to successfully increase EBF prevalence *via* the 2014–2018 National Breastfeeding Strategy to 28% (13). However, despite this increase EBF prevalence in Mexico is still below the world's average (44 vs. 50%) (14) and evidence shows that health inequities in breastfeeding promotion and protection are not narrowing. According to Unar-Mungía et al. (7), the largest increase in EBF was in Mexico City, in non-indigenous women, and in those belonging to high socio-economic status, while the States in the South of Mexico, region that concentrates the largest share of the country's 100 most marginalized municipalities (15), had the lowest or even no improvement in different breastfeeding indicators.

The lack of strong actions and policies for EBF protection and promotion has important implications for public health in terms of costs, morbidity, and mortality. Particularly, the need for regulation of milk formula commercialization and advertisement, training of health personnel, regulation of breastfeeding rooms in workspaces, maternity leave duration policies, protection in the informal sector, and establishment of penalties for breaching the International Code of Marketing of Breastmilk Substitutes are all areas that need attention, so that actions directed to promote and support breastfeeding become more successful (16).

### 1.2. Barriers and policies for breastfeeding protection

Among all barriers to EBF, in Mexico, the need of returning to work is considered one of the key factors for not breastfeeding overall and for early weaning among mothers who seek to breastfeed (17). Women's right to breastfeed has been ensured since 1974 in the United Mexican States Political Constitution. Even though there are many areas in the legal framework that need short-term action, particularly breastfeeding promotion and protection in the informal sector has not been addressed. Currently, legislation is mainly focused on ensuring that women can provide breastfeeding without this representing the loss of formal employment or remuneration, for a limited (not optimal) time. Also, these laws provide the basis for suitable nursing spaces where women can extract milk (2 times during a period of 30 min). Nevertheless, women in the informal sector are left out of these initiatives and this lack of social protection is another structural and societal barrier that interferes with women's ability and right to optimally breastfeed (18).

## 2. Unconditional cash transfers

Due to the above, there is a need to implement public policies directed at this vulnerable group, in which unconditional cash transfers (UCTs) could arise as a way to “level the playfield.” Cash transfers (CTs) programs are interventions aimed to address a key social determinant of health: income. Recent evidence shows that UCTs and Conditional Cash Transfers (CCTs) have positive impacts on a wide range of outcomes (19–21). However, unintended consequences have also been reported for CCTs such as exacerbation of social exclusion and creating opportunities for abuse of power (20). Operationally, conditionalities upon CCTs carries financial and administrative burdens, and certain behavioral requirements that may further complicate the success of the program in many LMICs, including Mexico (20).

On the other hand, UCTs programs are social and protection strategies that provide a small CTs to low-income families and communities where the only requirement to receive this financial support is to be subscribed to the benefit (22). And previous evidence suggests that UCTs for Mexican women working in the informal economy are economically feasible since in terms of costs they are similar to other programs already implemented in the country (23).

While CCTs programs have shown success over diverse nutrition-related outcomes, the short-lived window duration of time for EBF implies logistical difficulties and expenses that come with verification of these conditions, thus delaying transfers, and increasing the possibility that no timely transfers are made (24).

### 2.1. UCTs and health outcomes

There is a lack of evidence regarding UCTs and breastfeeding practices in LMICs. However, there is previous evidence about UCTs promoting positive health outcomes in maternal-child health. According to Durao et al. (24), UCTs in LMICs have shown high certainty evidence of improving food security, and low certainty of increasing dietary diversity and reducing stunting, as well as other nutrition-related outcomes. Also, Briaux et al. (25) reported that UCTs, in addition to community activities, improved children's linear growth in rural areas of Africa, while other studies (19) reported protective effects of UCTs by improving height for age, intake of animal food sources, household food insecurity, health-seeking behavior, delivery in health facilities and lowering the odds of low birthweight babies. For breastfeeding outcomes, Relton et al. (26) found that in areas with a low breastfeeding prevalence, providing financial incentives, conditional on the infant receiving any breast milk, improved breastfeeding rates.

Additionally, a relatively recent study reported a methodological framework to estimate the annual financial need of setting up maternity CTs for informally employed women in Mexico, reporting ranges between $87 million and $280 million dollars (18). This can be considered financially feasible since it is similar to social protection policies already implemented in the country directed to other population groups (23) and is considerably lower than the costs of not breastfeeding (18). Therefore, considering the logistical difficulties of CCTs, UCTs program for breastfeeding promotion and protection could be a promising policy in Mexico to improve children's health and development and to potentially enhance EBF prevalence. Also, it could get Mexico closer to achieving global nutrition targets and contribute to accomplishing children's good nutrition rights.

## 3. Discussion

Despite overall increases in EBF prevalence through many diverse policies, inequities in breastfeeding protection and promotion are still present in Mexico. One of the main barriers for EBF in Mexico and many LMICs is related to difficulties for women returning to work, and current legislation lacks policies to protect and promote better breastfeeding practices in the sector of the population that is the most unprotected, women working in the informal economy. These women are part of a considerably large population group who lack social protection measures and are subject to income instability, increasing their need to return to work, and thus challenging EBF practices. Policies should start supporting these groups in the population where EBF prevalence is not increasing, particularly vulnerable groups such as indigenous women and women in the southern states of the country.

Therefore, as a measure of social justice, we consider that CTs should be considered a plausible and adequate intervention to protect and promote breastfeeding in this population group without social protection. Even though there is favorable evidence of CCTs programs and breastfeeding and health-related outcomes, they entail administrative difficulties that, considering the very short window of support required for EBF and administrative costs, could jeopardize the success of the program. To our knowledge, there is no evidence of breastfeeding outcomes and UCTs in LMICs, however, there is evidence of positive outcomes in different health-related areas of UCTs programs, such as the experience in Togo (25) and Ecuador (27), as well as other LMICs (21). Also, considering that UCTs and CCTs have had similar health-related outcomes, it could also be the case for lactation.

However, considering that current evidence of UCTs on health outcomes comes from other LMICs, and that LMICs vary widely in EBF practices (28), as well as in structural, political, and community conditions that could impact the program, we consider the potential success resulting from a UCTs intervention in Mexico needs to be addressed in a pilot study. Mexico has a history of successfully implementing community-level programs to improve child nutrition-related outcomes such as PROGRESA/OPORTUNIDADES (29, 30), where similar communities with vulnerable populations would randomly receive the program at different times, thus allowing a comparison of the nutrition indicators between the early- vs late-implementation communities, generating evidence for a larger level implementation of the program. A pilot UCTs program that is aimed to improve breastfeeding practices for women in the informal economy could use a similar design, starting in most disadvantaged communities. Also, among starting points, we consider a monthly cash transfer would be suitable instead of a one-time transfer since they have shown to promote expenditure in basic household needs (31). Regarding inclusion criteria, all pregnant (3rd trimester) or lactating women without social protection provided by their work should be considered, which could probably be verified against social security databases. Moreover, we consider an indirect disbursement mechanism would be appropriate since a registry of informally working women could be a delicate subject. Fortunately, regarding the amount and duration of the program, results from Vilar-Compte et al. (18) support using the poverty line for reference and a duration of 6 months as feasible (from birth up to 6 months post-partum). Finally, it could also be useful to leverage other potential interventions alongside UCTs, such as counseling in primary healthcare (32), compliance of the Code of Marketing of Breastmilk Substitutes in the Mexican legislation, raising national awareness on breastfeeding (33), and engaging key stakeholders involved in breastfeeding policy and programming (34).

Therefore, we consider that a well-designed UCTs pilot study for women working in the informal economy should start to be considered in Mexico as a potentially appropriate intervention to overcome this contextual barrier. Also, with an adequate design and implementation, it could contribute to the generation of further evidence and work as a reference regarding the effect of UCTs programs in breastfeeding practices in LMICs with similar social and health issues.

## Author contributions

All authors listed have made a substantial, direct, and intellectual contribution to the work and approved it for publication.
